# Paraoxonase-2 Silencing Enhances Sensitivity of A375 Melanoma Cells to Treatment with Cisplatin

**DOI:** 10.3390/antiox9121238

**Published:** 2020-12-07

**Authors:** Roberto Campagna, Tiziana Bacchetti, Eleonora Salvolini, Valentina Pozzi, Elisa Molinelli, Valerio Brisigotti, Davide Sartini, Anna Campanati, Gianna Ferretti, Annamaria Offidani, Monica Emanuelli

**Affiliations:** 1 Department of Clinical Sciences, Polytechnic University of Marche, 60126 Ancona, Italy; rob_campagna@yahoo.com (R.C.); e.salvolini@univpm.it (E.S.); valentinapozzi81@gmail.com (V.P.); g.ferretti@univpm.it (G.F.); m.emanuelli@univpm.it (M.E.); 2 Department of Life and Environmental Sciences, Polytechnic University of Marche, 60131 Ancona, Italy; t.bacchetti@univpm.it; 3 Department of Clinical and Molecular Sciences, Polytechnic University of Marche, 60126 Ancona, Italy; molinelli.elisa@gmail.com (E.M.); valeriobrisigotti@hotmail.it (V.B.); a.campanati@univpm.it (A.C.); a.m.offidani@univpm.it (A.O.); 4 New York-Marche Structural Biology Center (NY-MaSBiC), Polytechnic University of Marche, 60131 Ancona, Italy

**Keywords:** melanoma, paraoxonase-2, chemotherapeutic drugs, cell proliferation, cell viability, oxidative stress

## Abstract

Melanoma represents the most aggressive skin cancer, being responsible for the majority of deaths related with these neoplasms. Despite chemotherapy represents a frontline approach for management of the advanced stages of the disease, it displayed poor response rates and short-term efficacy due to melanoma cell resistance. Therefore, the discovery of molecules that can be used for effective targeted therapy of melanoma is crucial. In this study, we evaluated the impact of paraoxonase-2 (PON2) silencing on proliferation, viability, and resistance to treatment of the A375 melanoma cell line with chemotherapeutic drugs dacarbazine (DTIC) and cisplatin (CDDP). Due to the enzymes ability to counteract oxidative stress, we also evaluated the effect of enzyme knockdown on reactive oxygen species (ROS) production in cells treated with CDDP. The data reported clearly demonstrated that PON2 knockdown led to a significant reduction of cell proliferation and viability, as well as to an enhancement of A375 sensitivity to CDDP treatment. Moreover, enzyme downregulation was associated with an increase of ROS production in CDDP-treated cells. Although further analyses will be necessary to understand how PON2 could influence melanoma cell metabolism and phenotype, our results seem to suggest that the enzyme may serve as an interesting molecular target for effective melanoma treatment.

## 1. Introduction

Cutaneous melanoma is the most aggressive form of skin neoplasm. Indeed, although it accounts for only 1% of all skin cancers, it is responsible for the majority of deaths associated with these tumors. Melanoma originates from malignant transformation of melanocytes and is characterized by an increasing incidence, mostly within western countries [1,2,3].

In terms of risk factors, a significant correlation between melanoma development and intermittent but intense exposure to UV radiation was found. Moreover, a family history of melanoma represents an important constitutional risk factor [4].

Time of detection compared with that of disease onset is a crucial aspect that greatly affects melanoma management and prognosis. In fact, surgical excision can be adopted as a curative intervention in early stage primary tumors only [1], while advanced disease is often associated with poor prognosis [5]. Melanoma cells display an intrinsic invasive nature, so even small-sized primary lesions are able to rapidly metastasize to multiple organs, thus leading to infaust outcomes. Given these features, the 5-year overall survival of patients affected with advanced stage melanoma is less than 15% [5].

In addition to conventional strategies, such as surgery and radiation therapy, chemotherapy, immunotherapy, and targeted therapy represent important approaches for the management of metastatic disease [6]. However, despite progress in research focused on cytotoxic, immunoactive, and targeted molecules, the median survival of patients suffering from advanced stage melanoma has not significantly improved [7].

Main chemotherapeutic drugs used for melanoma treatment include dacarbazine (DTIC) and temozolomide (TMZ), alkylating agents that induce a cytotoxic effect by inhibiting the DNA replication process [8]. Results obtained from several clinical trials demonstrated that around 12% of melanoma patients successfully respond to DTIC treatment and positive responses are durable in less than 2% subjects [6,8]. Studies aimed at evaluating the response induced by DTIC versus that exerted by TMZ concluded that both drugs display the same anti-neoplastic efficacy [6,7]. Carboplatin and cisplatin (CDDP) represent further cytotoxic compounds used to treat advanced-stage melanoma. They are able to form DNA crosslinks, thus inhibiting both replication and transcription. Similarly to DTIC and TMZ, CDDP administration leads to a 15% response rate [7,8]. Although chemotherapy plays a prominent role in management of subjects affected with late-stage disease, it shows a reduced long-term efficacy due to intrinsic or acquired resistance of melanoma cells [6].

In light of the above considerations, diagnostic delay and intrinsic melanoma cell aggressiveness represent important determinants of poor prognosis of this neoplasm. Therefore, the identification of molecular biomarkers that can be used for early detection and to setup targeted strategies for effective melanoma treatment are of the utmost importance.

The human paraoxonase (PON) gene family include three members: paraoxonase-1 (PON1), paraoxonase-2 (PON2), and paraoxonase-3 (PON3). Unlike PON1 and PON3, that are primarily expressed in the liver and subsequently secreted into the serum, PON2 is expressed in many tissues and localizes intracellularly upon translation [9]. Constitutive PON2 expression was found in both primary and immortalized endothelial cell lines, where it displays antioxidant properties [10]. Subsequent studies demonstrated that PON2 is also involved in the antioxidative response in the small intestine [11] and central nervous system [12].

Concerning enzyme intracellular localization, PON2 was found to be associated with the nuclear envelope [13], endoplasmic reticulum [13], mitochondria [14,15], and plasma membrane [16]. The ability of PON2 to counteract oxidative stress is strictly related to its efficiency in lowering production of reactive oxygen species (ROS) [15]. In mitochondria, the enzyme binds with high affinity to coenzyme Q10 within the inner membrane, thus leading to a reduction of superoxide anion release during the electron transport chain [14,15].

Many research groups have recently focused their interest on exploring the role played by PON2 in tumor cells, and enzyme upregulation was found in oral [17], bladder [18], pancreatic [19], ovarian [20], and gastric [21] cancer.

Data reported in our recent study clearly demonstrated PON2 overexpression in melanoma samples compared with that of control nevi. Moreover, a positive correlation between enzyme levels and important clinicopathological parameters related to tumor aggressiveness was found [22]. In the light of these results, the evaluation of PON2 as a molecular target for this cutaneous neoplasm deserved to be carried out, focusing on exploring the ability of the enzyme to participate to the mechanisms involved in melanoma cell sensitivity to chemotherapy. Therefore, this work aimed to evaluate the effect of shRNA-mediated PON2 silencing on phenotype of the human skin melanoma A375 cell line. In order to explore enzyme involvement in sensitivity of melanoma cells to chemotherapeutic drugs, such as DTIC and CDDP, cell proliferation, viability, and ROS production were determined before and after treatment with cisplatin.

## 2. Materials and Methods

### 2.1. Cell Line and Culture Conditions

The A375 (ATCC^®^ CRL-1619T) cell line (human malignant melanoma) was obtained from the American Type Culture Collection. Cells were grown in Dulbecco’s Modified Eagle’s Medium containing 4.5 g/L glucose, supplemented with 2 mM L-glutamine, 10% fetal bovine serum, and 50 µg/mL gentamicin, at 37 °C with 5% CO_2_.

### 2.2. shRNA-Mediated Gene Silencing of PON2

To induce PON2 silencing, 4.0 × 10^4^ A375 cells/well were seeded in 24-well plates the day before transfection. The plasmids (0.5 µg/well) encoding shRNA targeted to PON2 (pLKO.1-647) or empty vectors (pLKO.1-puro) were utilized to transfect 90% confluent cells, whereas control cells were treated exclusively with transfection reagent (mock). The transfection procedure was performed using FuGENE HD Transfection Reagent (Promega, Madison, WI, USA), according to the manufacturer’s instructions.

Forty-eight hours after the transfection procedure, the medium was removed and replaced with a standard one, supplemented with 1 μg/mL puromycin to allow for the selection of cellular clones downregulating PON2. For all consequent experiments, puromycin-resistant cells were grown in complete medium supplemented with puromycin. The efficiency of PON2 gene silencing in A375 cells was assessed by Real-Time PCR and Western blot analysis.

### 2.3. Real-Time PCR

For Real-Time PCR analysis, cell pellets (1.0 × 10^6^) were homogenized in a lysis buffer and total RNA was collected using the SV Total RNA Isolation System (Promega, Madison, WI, USA) according to manufacturer’s instructions. RNA was spectrophotometrically evaluated (260 nm and 280 nm) for quality and quantity. Then, 2 µg of total RNA were reverse transcribed using M-MLV Reverse Transcriptase (Promega), utilizing random primers (60 min at 37 °C). Real-Time PCR was performed using the CFX96 Real-Time PCR Detection System (Bio-Rad Laboratories, Hercules, CA, USA) and SsoFast EvaGreen Supermix (Bio-Rad Laboratories, Hercules, CA, USA). The cDNA that was generated as described above was utilized as a template. The nucleotide sequences of forward and reverse primers, used for Real-Time PCR analyses, were the following: 5′-TCGTGTATGACCCGAACAATCC-3′ and 5′-AACTGTAGTCACTGTAGGCTTCTC-3′ for PON2, and 5′-TCCTTCCTGGGCATGGAGT-3′ and 5′-AGCACTGTGTTGGC GTACAG-3′ for β-actin. Thermal protocol included initial denaturation, followed by 40 cycles of 95 °C for 30 s and 58 °C for 30 s. The expression level of PON2 was expressed as ΔCt value, where ΔCt = Ct (PON2) − Ct (β-actin). Fold changes in relative gene expression were calculated by the 2^-;ΔΔCt^ method. Each experiment was performed in triplicate and independently repeated three times.

### 2.4. Western Blot Analysis

A375 cell pellets (2 × 10^6^ cells) were lysed with 100 μL of lysis buffer (50 mM HEPES, pH 7.9, containing 150 mM NaCl, 0.5% Triton X-100, 1 mM phenylmethylsulphonyl fluoride and 2 μg/mL aprotinin) and homogenized through a 30 gauche syringe needle. Homogenate was centrifuged at 12,000× *g* for 15 min at 4 °C and the supernatant was collected. Samples containing 50 μg protein were subjected to 12.5% sodium dodecyl sulfate-polyacrylamide gel electrophoresis and transferred to polyvinylidene fluoride membranes.

Blots were first subjected to a blocking procedure and then incubated with primary and secondary antibodies. The first incubation was performed overnight at 4 °C with polyclonal anti-PON2 antibodies produced in rabbit (Sigma-Aldrich, St. Louis, MO, USA) at 1:500 dilution. Subsequently, membranes were incubated for 1 h at room temperature with 1:150,000 diluted horseradish peroxidase (HRP)-conjugated goat anti-rabbit IgG (Sigma-Aldrich, St. Louis, MO, USA). SuperSignal West Femto Maximum Sensitivity Substrate (Thermo Fisher Scientific, Waltham, MA, USA) was used to reveal PON2 protein signals. Chemiluminescent bands were then acquired using a ChemiDoc XRS+ System (Bio-Rad Laboratories, Hercules, CA, USA), and related signal intensity was quantified using Image Lab Software (Bio-Rad Laboratories, Hercules, CA, USA). Each experiment was performed in triplicate and independently repeated three times.

### 2.5. Cell Proliferation Assay

Cell proliferation was evaluated by trypan blue exclusion assay [23] on A375 cells seeded on six-well plates (3 × 10^5^ cells/well). Cells were maintained in serum-free medium for 24 h. At timepoint 0 h, medium was replaced with a standard one containing 10% FBS. For each timepoint, cells were harvested by 500 µL trypsin and centrifuged at 300× *g* for 3 min. Pellets were resuspended in 1 mL of complete medium and added to trypan blue. The number of viable cells (negative to trypan blue) was determined using Burker’s chamber. Each experiment was performed in triplicate and independently repeated three times.

### 2.6. Chemotherapeutic Treatment

A375 cells downregulating PON2, as well as controls, were seeded in 96-well plates (2.0 × 10^3^ cells/well). The day after seeding, cells were treated with CDDP or DTIC. A wide range of concentrations for both compounds was explored (0.1–8 µM and 10–200 µg/mL, respectively) prior to use. Selected concentrations were in accordance with previous studies [24,25]. DTIC was dissolved in HCl (2 mM final concentration), while CDDP was resuspended in complete medium.

### 2.7. MTT Assay

Cell viability was evaluated using a colorimetric assay with 3-(4,5-dimethylthiazol-2-yl)-2,5-diphenyl tetrazolium bromide (MTT), at different time points (0, 24, 48, and 72 h) in the presence or absence of DTIC and CDDP.

A375 cells were seeded in 96-well plates (2.0 × 10^3^ cells/well). Cells were allowed to attach overnight, and cell viability was evaluated by measuring the conversion of tetrazolium salt MTT to formazan crystals. Then, 10 μL of MTT reagent (5 mg/mL in phosphate buffered saline) was dissolved in 120 μL complete medium and added to the cells (100 μL/well). After incubation for 4 h at 37 °C, the medium was discarded and 200 μL of 2-propanol were added. The reaction product was quantified by measuring the absorbance at 540 nm using an ELISA plate reader. Experiments were repeated three times. Results were expressed as percentage of the control (0 h time point) and presented as mean values ± standard deviation of three independent experiments performed in triplicate.

### 2.8. Detection of Intracellular Oxidative Stress

Intracellular oxidative stress was examined by monitoring the oxidation of 2′,7′-dichlorodihydrofluorescein diacetate (DCFH_2_-DA) (Sigma-Aldrich, St. Louis, MO, USA). DCFH_2_-DA is promptly taken up by cells and consequently de-esterified to 2′,7′-dichlorodihydrofluorescein (DCFH_2_), a compound that is further oxidized to dichlorofluorescein (DCF) by ROS, including hydrogen peroxide, or reactive nitrogen species, such as peroxynitrite. Cells were seeded on 96-well black plates with clear bottoms (2.0 × 10^3^ cells per well) and allowed to adhere overnight. Subsequently, the old medium was discarded and cells were incubated in the dark with DCFH_2_-DA (50 μM) for 45 min at 37 °C. The probe was added from a stock solution using DMSO, which was also added to the blank. After washing to remove extracellular DCFH_2_-DA, the fluorescence was measured by using a plate reader at Ex/Em = (485/535) nm. Experiments were repeated three times. Results were presented as mean values ± standard deviation of three independent experiments performed in triplicate.

### 2.9. Statistical Analysis

GraphPad Prism software (GraphPad Software, San Diego, CA, USA) was used to statistically analyze the data obtained. One-way analysis of variance (ANOVA) was adopted to evaluate differences among examined samples. Statistical significance was set at *p* < 0.05.

## 3. Results

### 3.1. Efficiency of PON2 shRNA-Mediated Knockdown in A375 cells

The A375 cell line was transfected as described in the Materials and Methods section. To estimate the efficiency of PON2 downregulation, mRNA and protein levels were evaluated by Real-Time PCR and Western blot analysis.

**Figure 1:**
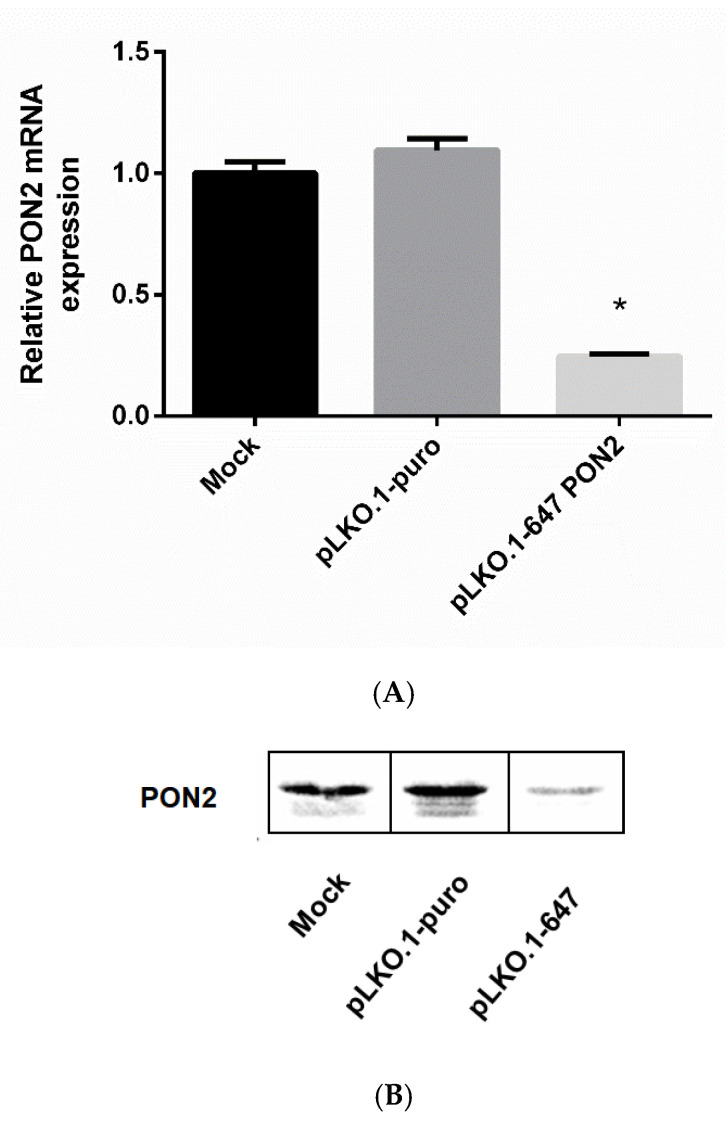

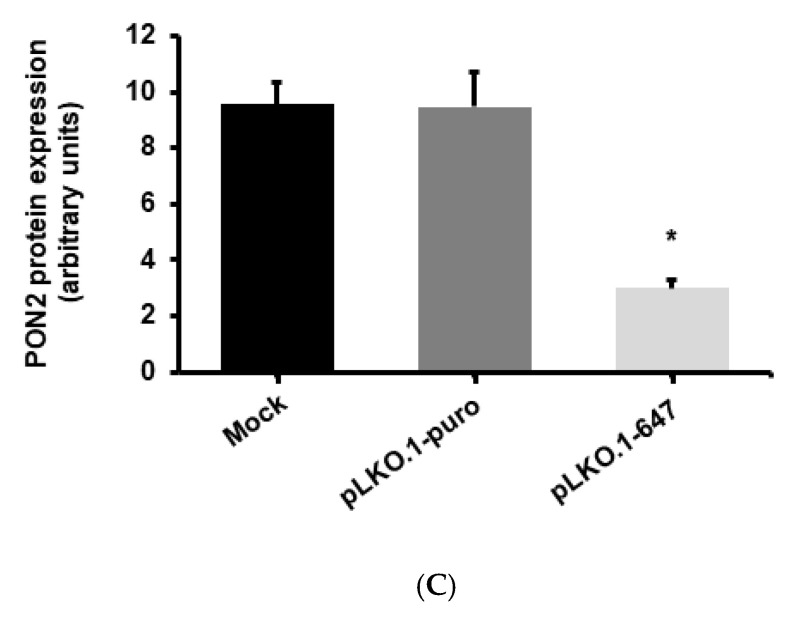
Evaluation of PON2 silencing. A375 cells were treated with a shRNA-coding plasmid against PON2 (pLKO.1-647), with empty vector (pLKO.1-puro), or with transfection reagent only (mock). PON2 expression was evaluated at mRNA and protein level by Real-Time PCR (panel **A**) and Western blot (panels **B**,**C**). Values are expressed as mean ± standard deviation (* *p* < 0.05).

Data obtained from Real-time PCR analyses revealed a significant (*p* < 0.05) decrease of PON2 mRNA levels in A375 cells treated with pLKO.1–647 (0.24 ± 0.01) with respect to controls (mock, 1.00 ± 0.05; pLKO.1-puro, 1.09 ± 0.04) (Figure 1A).

As shown in Figure 1B, illustrating the results of the Western blot analyses, a markedly decreased protein expression was reported for cells transfected with pLKO.1–647 in comparison with that of the mock and those treated with empty vectors. Moreover, densitometric analysis of immunoreactive bands demonstrated a significant (*p* < 0.05) enzyme downregulation in pLKO.1–647 (2.99 ± 0.31) compared with that of both mock (9.56 ± 0.76) and pLKO.1-puro (9.47 ± 1.23) samples (Figure 1C), thus confirming an effective PON2 gene silencing at the protein level.

### 3.2. Effect of PON2 Silencing on Cell Proliferation and Cell Viability

**Figure 2:**
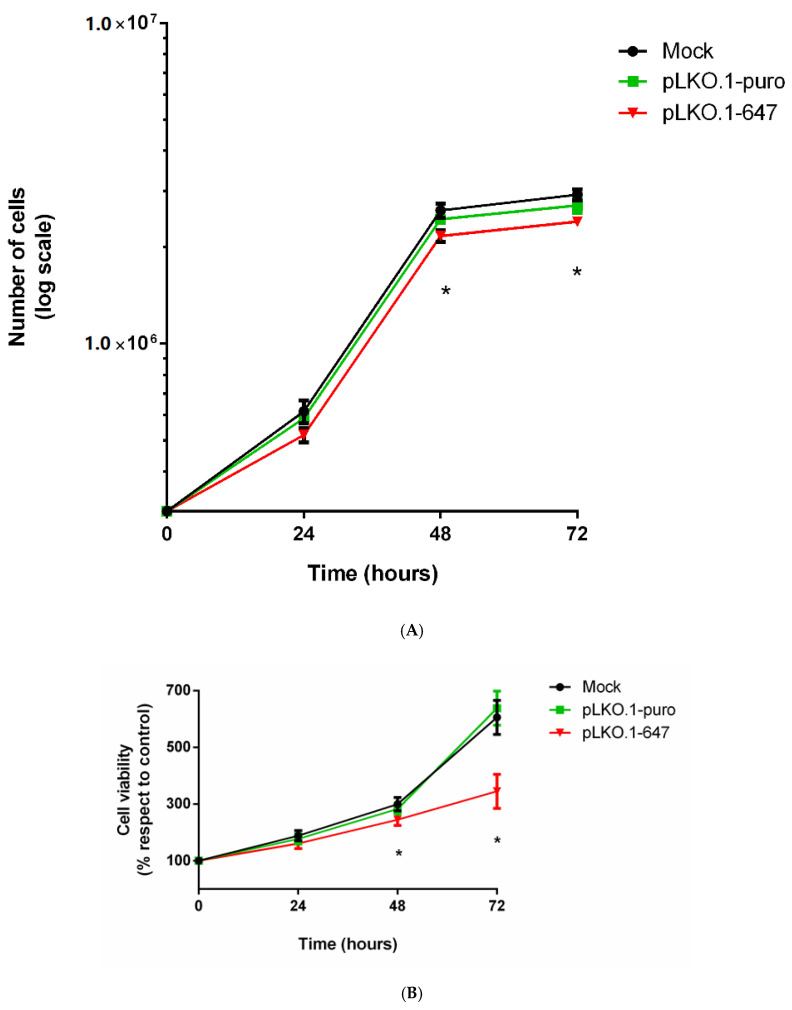
In vitro effect of PON2 silencing on cell proliferation and cell viability. Cell proliferation was analyzed by trypan blue exclusion assay in control samples (mock and pLKO.1-puro) and PON2 downregulating cells (pLKO.1-647) at 0, 24, 48, and 72 h (panel **A**). Cell viability was evaluated through MTT assay (panel **B**). Values are expressed as mean ± standard deviation (* *p* < 0.05).

To examine the role of PON2 in A375 tumor cell metabolism, and analyze the biological effect associated with enzyme downregulation, a vector encoding shRNA against PON2 was introduced into A375 cells, and proliferation and cell viability were then monitored. The effect of PON2 silencing on cell proliferation was evaluated by the trypan blue exclusion assay. As shown in Figure 2A, treatment with the pLKO.1-647 plasmid was able to significantly (*p* < 0.05) reduce cell growth compared with that of controls (mock and pLKO.1-puro).

Subsequently, the impact of PON2 downregulation on cell viability was evaluated by MTT assay. The results of this colorimetric assay were expressed as relative cell viability compared to control (absorbance at 0 h and equal to 100%). Enzyme downregulation resulted in significant (*p* < 0.05) reduced percentage values at timepoints 48 h and 72 h compared to that of cells transfected with empty vector or treated with transfection reagent only (Figure 2B).

### 3.3. Influence of PON2 Downregulation on Sensitivity of A375 Cells to Treatment with Cisplatin and Dacarbazine

**Figure 3:**
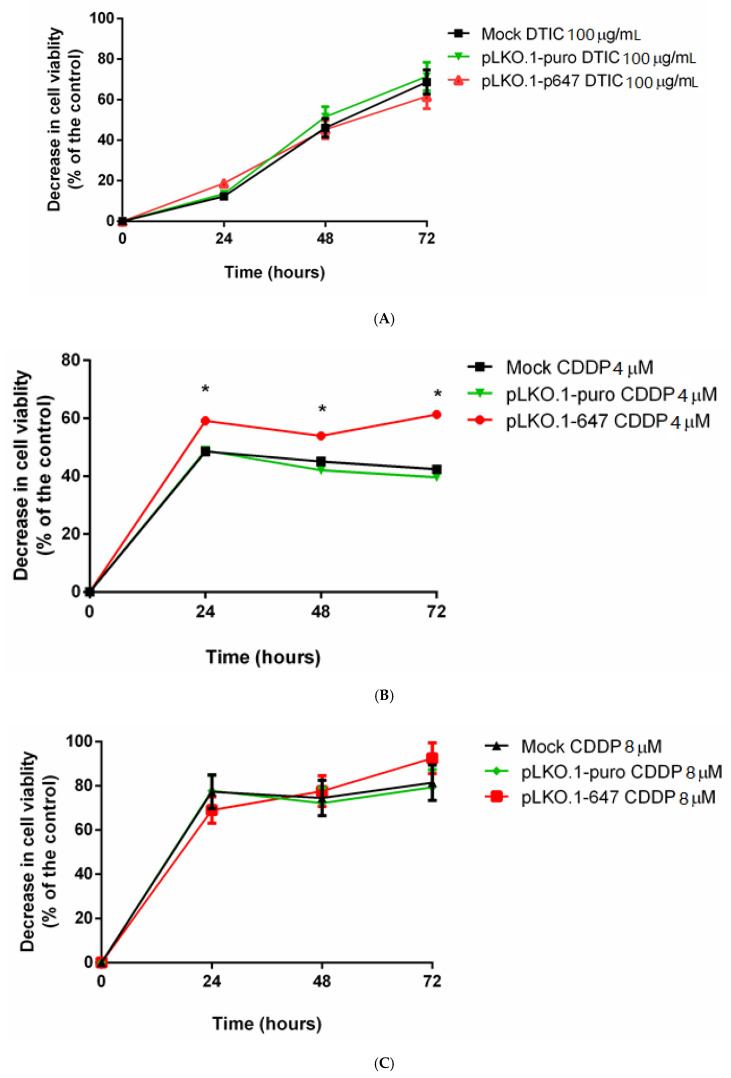
Effect of chemotherapeutic treatment on A375 cells. MTT assay was used to evaluate the effect of dacarbazine (DTIC) (100 µg/mL) (panel **A**) and cisplatin (CDDP) (4 and 8 µM) (panels **B**,**C**) on cell viability of mock, samples treated with empty vector (pLKO.1-puro), and PON2 downregulating cells (pLKO.1-647). Measurements were performed at different time points (0, 24, 48, and 72 h). All values are expressed as mean ± standard deviation (* *p* < 0.05).

MTT assay was used to evaluate the effect of treatment with the chemotherapeutic agent DTIC on cell viability of A375 cells downregulating PON2. Medium containing 2 mM HCl was determined to have no significant difference in cell proliferation compared to cells treated with complete medium only (data not shown). At all tested timepoints, the decrease of cell viability induced upon treatment with DTIC was similar among mock, pLKO.1-puro, and pLKO.1-647 samples (Figure 3A), thus demonstrating that PON2 silencing had no significant effect on A375 cell sensitivity to this compound.

Subsequently, MTT assay was performed on A375 downregulating PON2 treated with the chemotherapeutic agent CDDP at two different concentrations (4 and 8 µM). Interestingly, the decrease of cell viability upon treatment with 4 µM CDDP was markedly (*p* < 0.05) enhanced in PON2 downregulating A375 cells compared with that detected in mock and cells transfected with empty vector at all examined timepoints (Figure 3B). In contrast, treatment with 8 µM CDDP resulted in an excessive decrease of cell viability (Figure 3C).

### 3.4. Effect of PON2 Downregulation on ROS Production of A375 Cells Treated with Cisplatin

**Figure 4:**
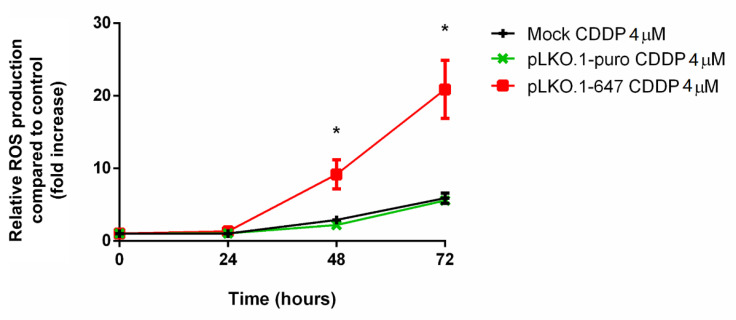
Intracellular reactive oxygen species (ROS) levels in A375 cells upon CDDP treatment. ROS levels were determined in mock, A375 transfected with empty vector (pLKO.1-puro), and PON2 downregulating cells (pLKO.1-647) upon treatment with CDDP (4 µM) at different time points (0, 24, 48, and 72 h). All values are expressed as the mean ± standard deviation (* *p* < 0.05).

To assess the effect of the induction of PON2 knockdown on the response to oxidative stress, intracellular ROS levels were evaluated in A375 cells after incubation with CDDP as well as in untreated cells. Without CDDP treatment, no changes in ROS production were observed in PON2 downregulating cells compared with that of controls (data not shown). Conversely, incubation with the chemotherapeutic drug led to a significant (*p* < 0.05) increase of ROS production in A375 cells transfected with pLKO.1-647 with respect to that of cells treated with empty vector (pLKO.1-puro) or with transfection reagent only (mock), at both 48 h and 72 h timepoints (Figure 4).

## 4. Discussion

The mainstay approach for management of patients suffering from late stage melanoma is represented by systemic therapy, which includes chemo-, immuno-, and targeted therapy. Over the last few decades, metastatic melanoma has been treated with cytotoxic chemotherapy, based on systemic administration of alkylating agents such as dacarbazine, temozolomide, platinum-derived compounds, and microtubule inhibitors, used alone or in combination [26].

DTIC is the only chemotherapeutic compound that has received approval from the Food and Drug Administration for clinical use in treatment of metastatic melanoma and is considered the “gold standard” for care of late stage disease. When used alone, it is able to induce an overall response rate ranging between 13% and 16%. However, most responses are partial, while complete responses emerge in 3% to 5% of patients and long-term remissions occur in less than 2% of treated subjects [26,27,28]. CDDP, which is widely used to treat a number of solid tumors, is also included among the anti-neoplastic drugs adopted in systemic cytotoxic chemotherapy for advanced-stage melanoma, even if its activity is modest. Indeed, CDDP, used as a single agent, induces an overall response rate in 15% of patients affected with metastatic disease, with 3 months median duration [7,8,28]. Therefore, it is evident that, although chemotherapy plays a leading role among strategies adopted to treat patients diagnosed with advanced melanoma, it does not represent an effective long-term intervention owing to resistance of the melanoma cells [6].

In the light of this evidence, the need to improve the efficacy of chemotherapeutic compounds, in terms of percentage increase of responders and achievement of long-term response, is crucial.

In our recent work, immunohistochemistry was performed to evaluate the expression level of PON2 enzyme in melanoma samples and control nevi. In addition, statistical analyses were performed in order to speculate on the potential correlations between enzyme levels and clinicopathological characteristics. The data obtained showed that PON2 was significantly upregulated in melanomas compared to that of controls. In addition, enzyme expression was found to be directly related with important prognostic parameters, such as Breslow thickness, Clark level, regression, mitoses, lymph node metastases, pT, and pathological stage, thus suggesting a potential role for PON2 as a biomarker for melanoma aggressiveness. [22]. Since PON2 overexpression is markedly enhanced in advanced lesions, which are known to be unsuccessfully treated with chemotherapy due to melanoma cell resistance, it was conceivable to hypothesize a potential enzyme contribution to molecular processes related to melanoma cell resistance to chemotherapeutic compounds.

In order to speculate on this hypothesis, in this study, we analyzed the role played by PON2 in melanoma cell metabolism by exploring the effect induced by enzyme knockdown on proliferation, viability, and chemosensitivity of the A375 melanoma cell line. Data reported clearly demonstrated that shRNA-mediated PON2 gene silencing was associated with a significant decrease of the proliferation rate and cell viability of A375 cells. In addition, enzyme downregulation led to a markedly enhanced sensitivity to treatment with cisplatin, while it did not seem to affect the response to dacarbazine. Moreover, PON2 knockdown was associated with enhancement of ROS production in CDDP-treated cells.

While the antioxidative effects exerted by PON2 have been described for a long time, only recently this enzyme was reported to be able to modulate execution of the apoptotic program in tumor cells. The activation of intrinsic apoptosis represents a major strategy adopted by cells to escape cancer formation. This pathway is mainly regulated by Bcl-2 protein members, due to their capacity to modulate mitochondrial pore opening and cytochrome C release, and by oxidative stress generated within mitochondria, that is able to remove cytochrome C from inner membrane-associated oxidized cardiolipin. In this light, PON2 activity, interacting with coenzyme Q10 and lowering of O_2_^−^ production, leads to the reduction of both cardiolipin peroxidation and cytochrome C release, thus significantly contributing to cancer cell resistance to apoptosis triggered by oxidative stress. The sum of these considerations strongly support data demonstrating that PON2 overexpression in tumor cells protects against treatment with chemotherapeutic drugs [29], mainly those acting by induction of the oxidative stress response.

DTIC is converted into its active form methyl-triazeno-imidazolecarboxamide (MTIC), which further yields a methyl carbonium ion. This latter acts as a highly reactive alkylating species, able to bind nucleophilic centers of DNA bases. Although the *N^1^*-position of guanine represents the most frequent alkylation site, *O^6^*-methylguanine (*O^6^*-meG), that recurs in less than 10% of cases, possesses a higher cytotoxic and mutagenic potential. During DNA replication, the presence of *O^6^*-meG, unremoved by the DNA repair systems, leads to the formation of *O^6^*-meG:T, with consequent generation of GC→AT mutation. Afterwards, the activity of the post-replication mismatch repair systems determines G2 phase cell cycle arrest, with subsequent induction of apoptosis or senescence [30]. Once it has entered the cell, CDDP undergoes hydrolytic displacement of chloride atoms, thus becoming a potent electrophilic species able to efficiently react with protein sulfhydryl groups and nitrogen atoms of nucleic acid bases. Within cancer cells, CDDP binds to the N7 of purine bases leading to DNA structural damage, with a subsequent cell division block and further induction of the apoptotic program. However, besides DNA damage, CDDP can also lead to the induction of cell death by promoting ROS production. Indeed, oxidative stress represents a fundamental condition through which CDDP cytotoxicity is exerted, and massive ROS release results in the apoptotic pathway activation. [31]. In the light of these considerations, it is clear how PON2 silencing had an impact on the efficacy of CDDP in reducing A375 cell viability, while it did not affect DTIC effectiveness.

Differences between effects induced upon treatment with CDDP at 4 and 8 µM may be interpreted based on comparison between related graphs. In A375 cells treated with 4 µM CDDP, the decrease of cell viability was already significantly different after 24 h incubation between PON2 downregulating cells (almost 60%) and that of controls (less than 50%). At the end of the time course, these differences were even enhanced, with a cell viability reduction of slightly over 60% for pLKO.1-647 and around 40% in both mock and pLKO.1-puro. In contrast, 8 µM CDDP led to higher percentage values of cell viability decrease (>65%) soon after 24 h incubation, indiscriminately for all samples. This trend was maintained until 72 h treatment, by which time the reduction of cell viability was ≥80%. It is evident that during incubation with 8 µM CDDP, the inhibitory stimulus induced on cell viability by treatment with the chemotherapeutic compound at this concentration was greatly higher than that exerted by PON2 downregulation, and therefore able to mask consequences brought about by enzyme expression decrease. Conversely, at 4 µM CDDP, the effects induced by molecular and pharmacological interventions were distinguishable, with enzyme knockdown being partly responsible for the enhancement of efficacy of drug treatment in reducing cell viability of A375 cells.

Although PON2 upregulation has been described in several tumors, the significance of enzyme overexpression and the effect induced by such dysregulation on cancer cell phenotypes (cell viability, migration and invasive capacity, metabolic rate, resistance to radio- and chemotherapy, etc.), remain only partly understood. In the last decade, a few studies have been carried out in order to elucidate these aspects.

Induction of PON2 gene silencing in gastric cancer cell lines revealed that enzyme downregulation was associated with a decrease of cell viability, migration, and invasive capacity [21]. Analogously, PON2 knockdown and overexpression were induced in the T24 BC cell line and the data reported clearly demonstrated that the enzyme was able to promote bladder cancer cell viability, migration, and resistance to treatment with the chemotherapeutic drugs cisplatin and gemcitabine [32]. Enzyme overexpression in the immortalized human vascular endothelial cell line EA.hy 926 treated with anthracycline doxorubicin was associated with reduction of ATP and a decrease and inhibition of caspase 3 activation. Moreover, apoptosis induced by treatment with staurosporine or actinomycin D was markedly decreased in EA.hy 926 cells upregulating PON2. Enzyme knockdown enhanced apoptosis-related death of chronic myeloid leukemia K562 cells receiving treatment with the Bcr-Abl tyrosine-kinase inhibitor imatinib, while the opposite effect was induced upon enzyme upregulation [33]. In oral cancer cell lines, short interfering (siRNA)-mediated PON2 silencing was found to be associated with enhancement of sensitivity to apoptotic damage caused by radiation treatment [17]. In pancreatic cancer cells, PON2 was able to facilitate glucose uptake and increase the efficiency of glucose metabolism by interacting with glucose transporter GLUT1. These traits are partly responsible for elevated pancreatic cancer cell aggressiveness, since they promote both cell growth and metastatic potential [19].

## 5. Conclusions

To date, this work is the first to analyze the role played by PON2 in melanoma, demonstrating enzyme involvement in those molecular events affecting cell viability and sensitivity to chemotherapeutic drugs. Although further studies will be necessary to widen our knowledge concerning processes featuring melanoma oncogenesis to which PON2 could participate, our data seem to suggest that the enzyme could serve as a promising molecular target for effective melanoma treatment.
